# Perioperative outcomes of atrial fibrillation in patients undergoing transcatheter aortic valve replacement: a national inpatient sample study

**DOI:** 10.1016/j.ijcha.2025.101688

**Published:** 2025-05-01

**Authors:** Adishwar Rao, Akriti Agrawal, Sivaram Neppala, Hitesh Bhatia, Ankur Singla, Albert Osei, Daniel Fabian, Fahd Syed, Saurabh Sharma

**Affiliations:** a Department of Internal Medicine, Guthrie Robert Packer Hospital, Sayre, PA, USA; b Department of Internal Medicine, UTHealth San Antonio, San Antonio, TX, USA; c Department of Internal Medicine, Northwest Health-Porter, Valparaiso, IN, USA; d Department of Cardiology, University of Pittsburgh Medical Center, Pittsburgh, PA, USA; e Department of Cardiology, Northwest Health, Valparaiso, IN, USA; f Department of Cardiology, Guthrie Robert Packer Hospital, Sayre, PA, USA

**Keywords:** Atrial fibrillation, Transcatheter aortic valve replacement, Tavr, Aortic stenosis, National inpatient sample, Perioperative outcomes

## Abstract

**Background:**

Transcatheter aortic valve replacement (TAVR) has transformed the management of patients with severe symptomatic aortic stenosis who are unfit for surgical aortic valve replacement. Atrial fibrillation is one of the most common arrhythmias observed in patients with aortic stenosis undergoing TAVR. Our study explores the impact of Afib on perioperative outcomes in patients undergoing TAVR.

**Methods:**

We queried the National Inpatient Sample from 2016 to 2021 for TAVR hospitalizations. We compared baseline features and outcomes between patients undergoing TAVR with and without Afib. We used the Pearson-Chi Square and Wilcoxon Rank Sum tests to compare categorical and continuous variables, respectively. We used multivariate logistic regression analysis to study the effect of Afib on outcomes.

**Results:**

Among 393,195 weighted hospitalizations for TAVR, 28.7 % had Afib. These patients were older (median age: 82 y vs. 79 y, p < 0.001) and predominantly males (58.6 % vs. 54.9 %, p < 0.001) compared to those without Afib. Multivariate analysis suggested that Afib was associated with greater odds of acute heart failure (aOR 1.27, p < 0.001), ventricular arrhythmia (aOR 1.33, p < 0.001), cardiogenic shock (aOR 1.38, p < 0.001), major adverse cardiac events (aOR 1.14, p = 0.002), acute kidney injury (aOR 1.27, p < 0.001), and acute bleeding (aOR 1.20, p < 0.001) compared to those without Afib in patients undergoing TAVR.

**Conclusion:**

Afib was associated with increased odds of acute heart failure, ventricular arrhythmia, cardiogenic shock, major adverse cardiac events, acute kidney injury, and acute bleeding in patients undergoing TAVR. However, the odds of in-hospital mortality in these patients were not significantly different from the group without Afib.

## Graphical abstract

**Figure:**
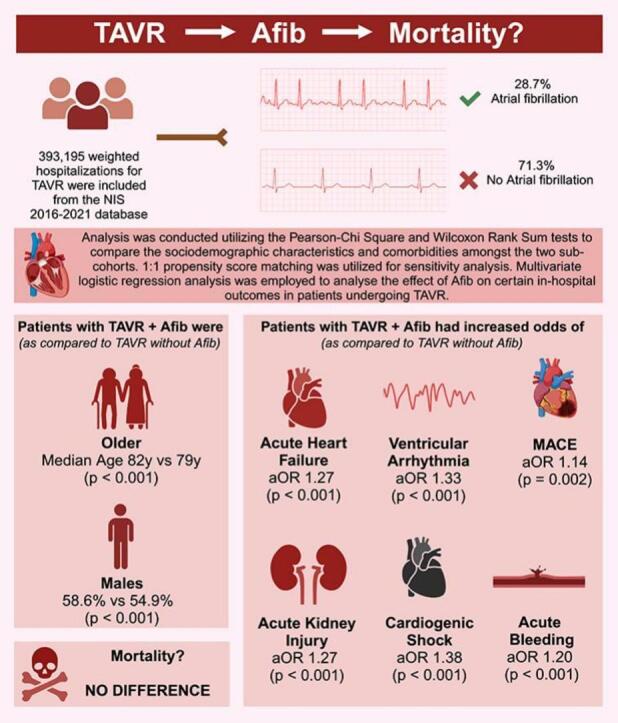

## 1 Introduction

Aortic stenosis is one of the most prevalent valvular diseases globally, which is associated with left ventricular outflow obstruction and may result in heart failure [1]. Transcatheter aortic valve replacement (TAVR) has transformed the management of severe aortic stenosis in patients not eligible for surgical aortic valve replacement (SAVR). Outcomes in TAVR have consistently proved to be non-inferior to conventional SAVR despite being significantly less invasive than SAVR [2]. Atrial Fibrillation (Afib) is the most common sustained arrhythmia, which complicates aortic stenosis, and studies suggest that new-onset Afib, a common complication of TAVR, is often associated with worse short and long-term clinical outcomes [3,4]. The timing of Afib has also been demonstrated as a critical parameter of concern in patients undergoing TAVR [5]. Notably, research from the STS/ACC TVT Registry has shown increased 1-year mortality and hospitalizations in patients developing new-onset Afib after the TAVR procedure [6]. However, most studies, including the landmark PARTNER trial, lack the assessment of the impact of Afib on in-hospital outcomes before discharge during the visit that TAVR was conducted [7]. Therefore, utilizing an extensive national database, we aim to assess the effect of Afib on perioperative outcomes, including in-hospital mortality, organ dysfunction, bleeding, and length of hospital stay, in patients undergoing TAVR.

## 2 Methods

### 2.1 Data source and cohort selection

In this study, we utilized hospital admissions data from the National Inpatient Sample (NIS) database provided by the Agency for Healthcare Research and Quality (AHRQ) under the Healthcare Cost and Utilization Project (HCUP) to conduct a cross-sectional study [8]. NIS is the largest all-payer database publicly available for outcomes research in the United States [9]. It uses a stratified sample of 20 percent discharges from all US community hospitals and estimates inpatient healthcare utilization, cost, and outcomes. We queried NIS for all hospitalizations in which TAVR was performed during the years 2016 to 2021 using International Classification of Diseases 10th Revision Procedural (ICD-10 PCS) codes. We excluded patients who were under 18 years of age or had missing data on age, sex, or mortality, which resulted in a sample size of 78,639 hospitalizations (weighted n = 393,195). We divided the sample into two groups based on the presence or absence of Afib in these patients. The ICD-10 codes used in this study are provided in Table S1.

### 2.2 Study variables and outcomes

In this study, we studied demographic characteristics, hospital metrics, comorbidities, and outcomes relating to TAVR patients with or without Afib. Age, sex assigned at birth, and race/ethnicity comprised the demographics where race was divided into White Americans, African Americans, Hispanics, Asians or Pacific Islanders, Native Americans, and Others. Hospital metrics included selecting patients who were hospitalized for two or more days in the hospital, as well as the cost burden associated with the hospitalization. We used the *TOTCHG* variable provided in the database and the consumer price index (CPI) statistics to calculate the inflation-adjusted economic burden for hospitalizations. Other hospital metrics included location and teaching status of the hospital, bed size of the hospital, region of the hospital, and the primary insurance used by the patients during their hospital stay.

We created comorbidity variables for multiple cardiovascular and non-cardiovascular comorbidities using their respective ICD-10 Clinical Modification (ICD-10 CM) codes. Cardiovascular comorbidities included hyperlipidemia, hypertension, chronic heart failure, obesity, history or current use of nicotine or cigarettes, pulmonary hypertension, and diabetes mellitus. We also accounted for previous cardio- or cerebrovascular incidents, such as prior myocardial infarction (MI) or prior stroke, and previous procedures or therapies used, such as prior percutaneous coronary intervention (PCI), coronary artery bypass graft (CABG) or pacemaker or defibrillator use. Non-cardiovascular comorbidities included in the study were chronic obstructive pulmonary disease (COPD), liver disease, obstructive sleep apnea (OSA), chronic kidney disease/end-stage kidney disease (CKD/ESRD), hypothyroidism, and nutritional anemia.

The primary outcome of the study was in-hospital mortality. The secondary outcomes were ST elevated MI (STEMI), acute heart failure (AHF), ventricular arrhythmia, cardiogenic shock (CS), mechanical circulatory support (MCS) use, major adverse cardiac events (MACE), acute kidney injury (AKI), acute stroke, and acute bleed. AHF included new-onset acute heart failure and acute-on-chronic heart failure. Ventricular arrhythmia comprised ventricular tachycardia, ventricular flutter, ventricular fibrillation, and reentrant ventricular arrhythmia. We computed MACE as a composite secondary outcome involving a documented ICD code for any of three events, STEMI, CS, or in-hospital mortality, during the same hospitalization as for the TAVR. MACE was not tracked post-discharge due to the nature of the database. Acute stroke included both acute ischemic and hemorrhagic strokes. Lastly, any patient who experienced hemorrhage secondary to cardiovascular graft insertion or who developed acute hemorrhagic anemia was considered to have an acute bleed.

### 2.3 Statistical analysis

The data was assessed for normality using histograms and QQ plots. Absolute counts and percentages were reported for categorical data and medians with their interquartile ranges were reported for continuous data. Pearson Chi-Square test was used to compare categorical variables while Wilcoxon Rank-Sum test was used to compare continuous variables. Multivariate analysis was performed using logistic regression models for each outcome and adjusted odds ratios (aOR) with their 95 % confidence intervals (95 % CI) and p-values were reported. Sensitivity analysis was conducted using 1:1 nearest neighbor propensity score matching with no replacement and outcomes were compared before and after propensity-score matching. Significance of the associations was assumed at p-value <0.05. All analyses for this study were weighted using the *DISCWT* variable provided in the NIS database. Stata statistical software by StataCorp, LLC, College Station, Texas, USA was used for the statistical analysis of this study [10].

### 2.4 Institutional Review Board (IRB) approval

IRB approval was not required for this study due to the publicly available nature of the deidentified data. Any counts under 11 were not reported to ensure compliance with HCUP’s policy to avoid identifying patients with rare diseases.

## 3 Results

### 3.1 Demographics and comorbidity burden

Our study analyzed baseline differences in demographics and comorbidities between TAVR patients with and without Afib *(*Table 1*)*. TAVR patients with Afib were older (median age: 82 y vs. 79 y, p < 0.001), had a higher proportion of males (58.6 % vs. 54.9 %, p < 0.001), and had significant racial/ethnic differences (White Americans: 90.8 % vs. 85.9 %; African Americans: 2.8 % vs. 4.6 %; Hispanics: 3.5 % vs. 5.4 %; Asians or Pacific Islanders: 1 % vs 1.5 %; Native Americans: 0.2 % vs 0.3 %; Others: 1.7 % vs. 2.3 %; p < 0.001) than TAVR patients without Afib.

**Table 1:** Descriptive statistics of patients undergoing TAVR compared between patients with Afib and without Afib.

	TAVR + Afib	TAVR only	p
N	112,945 (28.7 %)	280,250 (71.3 %)	
**Sex assigned at birth**			
Male	66,175 (58.6 %)	153,935 (54.9 %)	**<0.001**
Female	46,770 (41.4 %)	126,315 (45.1 %)	
**Age (y)**, Median (IQR)	82 (76–87)	79 (72–85)	**<0.001**
**Length of stay ≥ 3 days**	50,590 (44.8 %)	95,400 (34.0 %)	**<0.001**
**Race**			
White American	99,380 (90.8 %)	233,780 (85.9 %)	**<0.001**
African American	3,045 (2.8 %)	12,435 (4.6 %)	
Hispanic	3,810 (3.5 %)	14,645 (5.4 %)	
Asian or Pacific Islander	1,140 (1.0 %)	4,110 (1.5 %)	
Native American	235 (0.2 %)	860 (0.3 %)	
Other	1,840 (1.7 %)	6,375 (2.3 %)	
**Primary Insurance**			
Medicare	102,080 (90.5 %)	242,425 (86.6 %)	**<0.001**
Medicaid	1,135 (1.0 %)	4,750 (1.7 %)	
Private Insurance	7,210 (6.4 %)	25,450 (9.1 %)	
Self-pay	375 (0.3 %)	1,455 (0.5 %)	
No charge	20 (0.0 %)	85 (0.0 %)	
Other	1,985 (1.8 %)	5,800 (2.1 %)	
**Region of hospital**			
Northeast	25,380 (22.5 %)	63,060 (22.5 %)	**<0.001**
Midwest	28,320 (25.1 %)	62,695 (22.4 %)	
South	36,880 (32.7 %)	97,185 (34.7 %)	
West	22,365 (19.8 %)	57,310 (20.4 %)	
**Location/teaching status of hospital**			
Rural	1,555 (1.4 %)	3,705 (1.3 %)	0.224
Urban Non-Teaching	10,855 (9.6 %)	25,695 (9.2 %)	
Urban Teaching	100,535 (89.0 %)	250,850 (89.5 %)	
**Bed size of hospital**			
Small	8,475 (7.5 %)	21,155 (7.5 %)	0.624
Medium	24,695 (21.9 %)	60,200 (21.5 %)	
Large	79,775 (70.6 %)	198,895 (71.0 %)	
**Total Charge (Inflation-Adjusted)**, Median (IQR)	$195,914 ($143,202-$287,897)	$186,859 ($139,556-$271,135)	**<0.001**
**Total Cost**, Median (IQR)	$49,951 ($38,555-$65,102)	$47,755 ($37,231-$61,559)	**<0.001**
**Comorbidities**			
Hyperlipidemia	81,805 (72.4 %)	208,920 (74.5 %)	**<0.001**
Hypertension	103,370 (91.5 %)	249,840 (89.1 %)	**<0.001**
Chronic Heart Failure	90,185 (79.8 %)	189,675 (67.7 %)	**<0.001**
Prior MI	14,620 (12.9 %)	32,285 (11.5 %)	**<0.001**
Prior PCI	20,625 (18.3 %)	53,105 (18.9 %)	**0.047**
Prior CABG	16,095 (14.3 %)	35,150 (12.5 %)	**<0.001**
Prior Pacemaker or Defibrillator	20,945 (18.5 %)	23,035 (8.2 %)	**<0.001**
Obesity	23,075 (20.4 %)	58,890 (21.0 %)	0.093
Smoker/Nicotine user	44,250 (39.2 %)	113,265 (40.4 %)	**0.003**
COPD	27,265 (24.1 %)	57,390 (20.5 %)	**<0.001**
Obstructive Sleep Apnea	19,865 (17.6 %)	38,220 (13.6 %)	**<0.001**
Pulmonary Hypertension	24,925 (22.1 %)	36,315 (13.0 %)	**<0.001**
Prior Stroke	18,575 (16.4 %)	34,315 (12.2 %)	**<0.001**
Liver Disease	4,195 (3.7 %)	11,285 (4.0 %)	0.054
CKD/ESRD	46,005 (40.7 %)	89,545 (32.0 %)	**<0.001**
Diabetes Mellitus	42,280 (37.4 %)	105,695 (37.7 %)	0.495
Hypothyroidism	22,770 (20.2 %)	50,010 (17.8 %)	**<0.001**
Nutritional Anemia	6,675 (5.9 %)	12,720 (4.5 %)	**<0.001**

Abbreviations: TAVR, transcatheter aortic valve replacement, Afib, atrial fibrillation, p, p-value, IQR, interquartile range, MI, myocardial infarction, PCI, percutaneous coronary intervention, CABG, coronary artery bypass surgery, COPD, chronic obstructive pulmonary disease, CKD/ESRD, chronic kidney disease/end stage renal disease.

TAVR patients with Afib had higher proportions of comorbidities such as hypertension (91.5 % vs. 89.1 %, p < 0.001), chronic heart failure (79.8 % vs. 67.7 %, p < 0.001), COPD (24.1 % vs. 20.5 %, p < 0.001), OSA (17.6 % vs 13.6 %, p < 0.001), pulmonary hypertension (22.1 % vs. 13 %, p < 0.001), CKD/ESRD (40.7 % vs. 32 %, p < 0.001), hypothyroidism (20.2 % vs. 17.8 %, p < 0.001), and nutritional anemia (5.9 % vs 4.5 %, p < 0.001) than those without Afib.

TAVR patients with Afib also had higher proportions of prior cardio- or cerebrovascular incidents and procedures: prior MI (12.9 % vs 11.5 %, p < 0.001), prior pacemaker or defibrillator placement (18.5 % vs. 8.2 %, p < 0.001), prior CABG (14.3 % vs. 12.5 %, p < 0.001), and prior stroke (16.4 % vs. 12.2 %, p < 0.001) than patients without Afib. In contrast, TAVR patients with Afib had lower proportions of hyperlipidemia (72.4 % vs. 74.5 %, p < 0.001), smokers or nicotine users (39.2 % vs. 40.4 %, p = 0.003), and patients who underwent prior PCI (18.3 % vs. 18.9 %, p = 0.047) than TAVR patients without Afib.

### 3.2 Hospital metrics

Medicare was the largest insurance provider for TAVR patients with or without Afib, but those with Afib had a higher proportion of Medicare utilization (90.5 % vs. 86.6 %, p < 0.001) than those without Afib. A greater proportion of TAVR patients with Afib had a duration of hospital stay ≥ 3 days (44.8 % vs. 34 %, p < 0.001) than those without Afib. TAVR patients with Afib incurred higher total charges (median charge: $195,914 vs. $186,859; p < 0.001) and total costs (median cost: $49,951 vs. $47,755, p < 0.001) during hospitalization than TAVR patients without Afib.

### 3.3 Outcomes

**Fig. 1:**
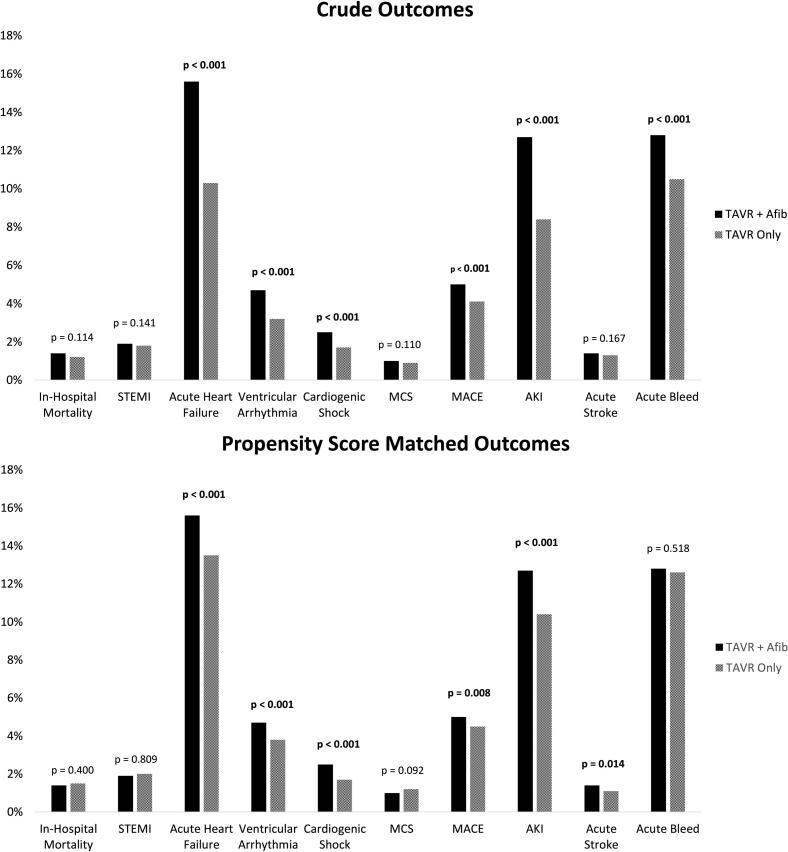
Crude and Propensity Score Matched Outcomes in patients undergoing TAVR with or without Afib Abbreviations: TAVR, transcatheter aortic valve replacement, Afib, atrial fibrillation, p, p-value, STEMI, ST elevated myocardial infarction, MCS, mechanical circulatory support, MACE, major adverse cardiac events, AKI, acute kidney injury.

Results from crude estimates suggest that TAVR patients with Afib had higher proportions of AHF (15.6 % vs. 10.3 %, p < 0.001), ventricular arrhythmia (4.7 % vs. 3.2 %, p < 0.001), CS (2.5 % vs. 1.7 %, p < 0.001), MACE (5 % vs. 4.1 %, p < 0.001), AKI (12.7 % vs. 8.4 %, p < 0.001), and acute bleed (12.8 % vs. 10.5 %, p < 0.001) than TAVR patients without Afib *(*Fig. 1*, Table S2)*.

Upon matching on demographics and comorbidities, propensity matched estimates suggest similar results with higher proportions of AHF (15.6 % vs. 13.5 %, p < 0.001), ventricular arrhythmia (4.7 % vs. 3.8 %, p < 0.001), CS (2.5 % vs. 1.7 %, p < 0.001), MACE (5 % vs. 4.5 %, p = 0.008), AKI (12.7 % vs. 10.4 %, p < 0.001), and acute stroke (1.4 % vs. 1.1 %, p = 0.014) in TAVR patients with Afib compared to those without Afib.

**Fig. 2:**
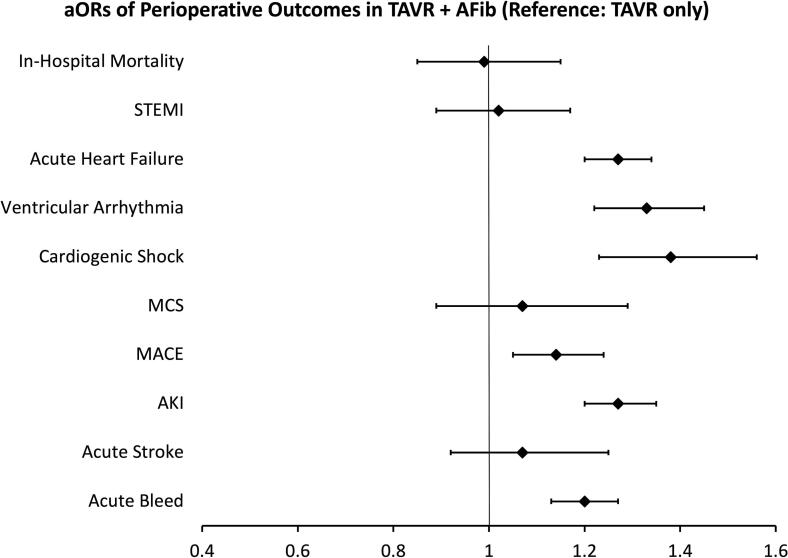
Forest plot from a multivariate analysis in TAVR patients with Afib (reference: TAVR patients without Afib) Abbreviations: aOR, adjusted odds ratio, TAVR, transcatheter aortic valve replacement, Afib, atrial fibrillation, STEMI, ST elevated myocardial infarction, MCS, mechanical circulatory support, MACE, major adverse cardiac events, AKI, acute kidney injury.

Lastly, results from multivariate logistic regression models for outcomes, while adjusting for demographics and comorbidities, suggest that Afib in TAVR patients was associated with higher odds of AHF (aOR 1.27, p < 0.001), ventricular arrhythmia (aOR 1.33, p < 0.001), CS (aOR 1.38, p < 0.001), MACE (aOR 1.14, p = 0.002), AKI (aOR 1.27, p < 0.001), and acute bleed (aOR 1.20, p < 0.001) compared to TAVR patients without Afib *(*Fig. 2*, Table S3, Table S4)*.

## 4 Discussion

Our study included 393,195 weighted hospitalizations for TAVR. 112,945 (28.7 %) patients had Afib, while 280,250 (71.3 %) did not have Afib. We analyzed the effects of Afib on perioperative outcomes in these patients. Our results suggest that in patients undergoing TAVR, Afib is associated with higher odds of AHF, ventricular arrhythmia, CS, MACE, AKI, and acute bleeding but not in-hospital mortality.

Literature suggests that Afib at discharge is associated with greater mortality in patients undergoing TAVR within five years (35.6 % vs 27.2 %, p < 0.01) [11]. Notably, our results suggest that perioperative mortality in patients undergoing TAVR with a history of Afib or new-onset Afib is similar to those without Afib, despite patients with Afib being at greater odds of multiple perioperative complications. These findings are consistent with a recent study based upon national data from a decade ago [12]. While our understanding of the effects of Afib has advanced tremendously in the last decade, increased long-term mortality in patients who underwent TAVR despite similar perioperative mortality suggests the persistence of therapeutic lacunae in the management of these patients post-discharge.

Results from our study suggest that patients undergoing TAVR with concomitant Afib have greater odds of AHF. While previous studies have described the deleterious impact of new-onset Afib on the development of AHF in patients undergoing TAVR (hazard ratio [HR] 1.98, p < 0.01), our data suggests that Afib is associated with increased odds of AHF, irrespective of the onset of Afib [5,13]. Moreover, our study also suggested that Afib was associated with greater odds of CS in patients undergoing TAVR. New onset Afib patients have traditionally been more predisposed to the development of AKI while undergoing TAVR, even with lower amounts of contrast compared to patients in sinus rhythm [11]. Our study supports this claim and broadens its applicability to not just new-onset Afib but also chronic and paroxysmal Afib, as Afib in patients undergoing TAVR was associated with significantly higher odds of AKI.

Current evidence on the length of hospital stay in patients undergoing TAVR suggests that over half of the patients are discharged within 72 h (or three days) of hospitalization [14]. Our study further explored whether a difference exists in the proportion of patients staying for three or more days based on the presence or absence of Afib. We found that among patients undergoing TAVR, the proportion of those hospitalized for three or more days with Afib was significantly higher than those without Afib. This difference also suggests that, compared to those without Afib, concomitant Afib in patients undergoing TAVR may contribute towards a higher economic burden on the patients and the healthcare system.

## 5 Study limitations

Our study involved a large sample size and provided greater insights into Afib in patients undergoing TAVR. However, it was subject to several limitations. First, the type of data we used limited us to conduct a cross-sectional study and did not allow us to assess the temporality of the outcomes of our study. Second, the lack of a patient linkage variable to track data across hospitalizations over the years restricted us from performing long-term follow-up analyses for trends in our study outcomes. Third, NIS is a billing database subject to administrative and coding errors, which may inevitably skew the actual association between our variables. Lastly, although we used propensity score-matching and logistic regression to minimize confounding factors, some unknown and unaddressed biases may remain. Despite these shortcomings, the large sample size used in the study strengthens the associations assessed and provides supportive evidence to the existing literature on Afib in patients undergoing TAVR.

## 6 Conclusion

In conclusion, results from the NIS database suggest that patients undergoing TAVR who also have Afib generally have worse perioperative outcomes such as AHF, ventricular arrhythmia, CS, MACE, AKI, and acute bleeding than those without Afib. Afib contributing to worse short-term outcomes in this subgroup of patients is still debatable as these patients were sicker at baseline than the comparison group. With Afib being a common arrhythmia encountered in clinical practice and an uptrend in TAVR procedures performed these days, prospective studies should be conducted involving multiple institutions to assess the relationship between Afib and perioperative outcomes in these patients further.

## Funding

The authors received no grants for the study.

## CRediT authorship contribution statement

**Adishwar Rao:** Writing – review & editing, Writing – original draft, Visualization, Validation, Methodology, Investigation, Formal analysis, Data curation, Conceptualization. **Akriti Agrawal:** Writing – review & editing, Writing – original draft, Validation, Investigation, Formal analysis, Data curation. **Sivaram Neppala:** Writing – review & editing, Writing – original draft, Validation, Data curation. **Hitesh Bhatia:** Writing – review & editing, Validation, Conceptualization. **Ankur Singla:** Writing – review & editing, Validation, Project administration, Data curation. **Albert Osei:** Writing – review & editing, Validation, Project administration, Data curation. **Daniel Fabian:** Writing – review & editing, Validation, Investigation, Conceptualization. **Fahd Syed:** Writing – review & editing, Validation, Supervision, Project administration, Conceptualization. **Saurabh Sharma:** Writing – review & editing, Visualization, Validation, Supervision, Methodology, Data curation, Conceptualization.

## Declaration of competing interest

The authors declare that they have no known competing financial interests or personal relationships that could have appeared to influence the work reported in this paper.
