# Supplementary material for: Perioperative outcomes of atrial fibrillation in patients undergoing transcatheter aortic valve replacement: a national inpatient sample study
Source: Int J Cardiol Heart Vasc. 2025 May 1;59:101688. doi: 10.1016/j.ijcha.2025.101688 (PMC12084496; doi:10.1016/j.ijcha.2025.101688)
Supplement: Supplementary Data 1 [file mmc1.docx]

**Supplementary File**

**Title**: Perioperative Outcomes of Atrial Fibrillation in Patients Undergoing Transcatheter Aortic Valve Replacement: A National Inpatient Sample Study

**Authors:** Adishwar Rao, Akriti Agrawal, Sivaram Neppala, Hitesh Bhatia, Ankur Singla, Albert Osei, Daniel Fabian, Fahd Syed, Saurabh Sharma

**Supplementary materials Legend:**

1. Table S1: ICD-10 CM and ICD-10 PCS codes used in the study.
2. Table S2: Comparison of crude estimates of primary and secondary outcomes with propensity-matched estimates.
3. Table S3: Multivariate analysis of primary and secondary outcomes assessing the effect of Afib in TAVR patients compared to those TAVR patients without Afib
4. Table S4: Variables adjusted for in the logistic regression model and propensity-score matching.

**Table S1: ICD-10 CM and ICD-10 PCS codes used in the study**

| **Variable** | **ICD-10 CM or PCS code** |
| --- | --- |
| **Cohort** | |
| TAVR | 02RF37H 02RF38H 02RF3JH 02RF3KH 02RF37Z 02RF38Z 02RF3JZ 02RF3KZ X2RF332 |
| Afib | I480 I481 I4811 I4819 I482 I4820 I4821 |
| **Comorbidities** | |
| Hyperlipidemia | E785 E7800 E781 E782 E783 E784 E7849 E780 E7800 E7801 |
| Hypertension | I10 I119 I150 I151 I152 I158 I159 I160 I161 I169 |
| Chronic Heart Failure | I501 I5020 I5021 I5022 I5023 I5030 I5031 I5032 I5033 I5040 I5041 I5042 I5043 I50810 I50811 I50812 I50813 I50814 I5082 I5083 I5084 I5089 I509 |
| Prior MI | I252 |
| Prior PCI | Z955 |
| Prior CABG | I252 I25700 I25701 I25708 I25709 I25710 I25711 I25718 I25719 I25720 I25721 I25728 I25729 I25730 I25731 I25738 I25739 I25790 I25791 I25798 I25799 I25810 |
| Prior Pacemaker or Defibrillator | Z950 Z95810 |
| Obesity | E6601 E6609 E661 E662 E668 E669 Z6830 Z6831 Z6832 Z6833 Z6834 Z6835 Z6836 Z6837 Z6838 Z6839 Z6841 Z6842 Z6843 Z6844 Z6845 O99210 O99211 O99212 O99213 O99214 O99215 |
| Smoker/Nicotine user | Z720 O99330 O99331 O99333 O99334 O99335 F17200 F17201 F17203 F17208 F17209 F17210 F17211 F17213 F17218 F17219 F17220 F17221 F17223 F17228 F17229 F17290 F17291 F17293 F17298 F17299 Z87891 |
| COPD | J410 J411 J418 J42 J430 J431 J432 J438 J439 J440 J441 J449 |
| Obstructive Sleep Apnea | G4733 |
| Pulmonary Hypertension | I270 I272 I2720 I2721 I2722 I2723 I2724 I2729 |
| Prior Stroke | Z8673 I6930 I6931 I69310 I69311 I69312 I69313 I69314 I69315 I69318 I69319 I69320 I69321 I69322 I69323 I69328 I69331 I69332 I69333 I69334 I69339 I69341 I69342 I69343 I69344 I69349 I69351 I69352 I69353 I69354 I69359 I69361 I69362 I69363 I69364 I69365 I69369 I69390 I69391 I69392 I69393 I69398 |
| Liver Disease | B180 B181 B182 B188 B189 K700 K7010 K7011 K702 K7030 K7031 K7040 K7041 K709 K710 K7110 K7111 K712 K713 K714 K7150 K7151 K716 K717 K718 K719 K7200 K7201 K7210 K7211 K7290 K7291 K730 K731 K732 K738 K739 K740 K7400 K7401 K7402 K741 K742 K743 K744 K745 K7460 K7469 K750 K751 K752 K753 K754 K7581 K7589 K759 K760 K761 K762 K763 K764 K765 K766 K767 K7681 K7689 K769 K77 |
| CKD/ESRD | N181 N182 N183 N184 N185 N186 N189 I120 I129 I130 I1310 I1311 I132 D631 E0822 E1022 E1122 Z992 |
| Diabetes Mellitus | E1010 E1011 E1021 E1022 E1029 E10311 E10319 E10321 E103211 E103212 E103213 E103219 E10329 E103291 E103292 E103293 E103299 E10331 E103311 E103312 E103313 E103319 E10339 E103391 E103392 E103393 E103399 E10341 E103411 E103412 E103413 E103419 E10349 E103491 E103492 E103493 E103499 E10351 E103511 E103512 E103513 E103519 E103521 E103522 E103523 E103529 E103531 E103532 E103533 E103539 E103541 E103542 E103543 E103549 E103551 E103552 E103553 E103559 E10359 E103591 E103592 E103593 E103599 E1036 E1037X1 E1037X2 E1037X3 E1037X9 E1039 E1040 E1041 E1042 E1043 E1044 E1049 E1051 E1052 E1059 E10610 E10618 E10620 E10621 E10622 E10628 E10630 E10638 E10641 E10649 E1065 E1069 E108 E109 E1100 E1101 E1110 E1111 E1121 E1122 E1129 E11311 E11319 E11321 E113211 E113212 E113213 E113219 E11329 E113291 E113292 E113293 E113299 E11331 E113311 E113312 E113313 E113319 E11339 E113391 E113392 E113393 E113399 E11341 E113411 E113412 E113413 E113419 E11349 E113491 E113492 E113493 E113499 E11351 E113511 E113512 E113513 E113519 E113521 E113522 E113523 E113529 E113531 E113532 E113533 E113539 E113541 E113542 E113543 E113549 E113551 E113552 E113553 E113559 E11359 E113591 E113592 E113593 E113599 E1136 E1137X1 E1137X2 E1137X3 E1137X9 E1139 E1140 E1141 E1142 E1143 E1144 E1149 E1151 E1152 E1159 E11610 E11618 E11620 E11621 E11622 E11628 E11630 E11638 E11641 E11649 E1165 E1169 E118 E119 E1300 E1301 E1310 E1311 E1321 E1322 E1329 E13311 E13319 E13321 E133211 E133212 E133213 E133219 E13329 E133291 E133292 E133293 E133299 E13331 E133311 E133312 E133313 E133319 E13339 E133391 E133392 E133393 E133399 E13341 E133411 E133412 E133413 E133419 E13349 E133491 E133492 E133493 E133499 E13351 E133511 E133512 E133513 E133519 E133521 E133522 E133523 E133529 E133531 E133532 E133533 E133539 E133541 E133542 E133543 E133549 E133551 E133552 E133553 E133559 E13359 E133591 E133592 E133593 E133599 E1336 E1337X1 E1337X2 E1337X3 E1337X9 E1339 E1340 E1341 E1342 E1343 E1344 E1349 E1351 E1352 E1359 E13610 E13618 E13620 E13621 E13622 E13628 E13630 E13638 E13641 E13649 E1365 E1369 E138 E139 |
| Hypothyroidism | E030 E031 E032 E033 E034 E035 E038 E039 |
| Nutritional Anemia | D500 D501 D508 D509 D510 D511 D512 D513 D518 D519 D520 D528 D529 D521 D530 D531 D532 D538 D539 |
| **Outcomes** | |
| STEMI | I2101 I2102 I2109 I2111 I2119 I2121 I2129 I213 I214 |
| AHF | I5021 I5023 I5041 I5043 I50811 I50813 |
| Ventricular Arrhythmia | I470 I472 I4901 I4902 |
| CS | R570 |
| MCS | 5A02110 5A02210 5A0211D 02HA3RZ 5A02116 5A0221D 5A1522F 5A1522G 5A1522H 5A15A2F 5A15A2G 5A15A2H 5A15223 |
| AKI | N170 N171 N172 N178 N179 N19 |
| Acute Stroke | I6300 I63011 I63012 I63013 I63019 I6302 I63031 I63032 I63033 I63039 I6309 I6310 I63111 I63112 I63113 I63119 I6312 I63131 I63132 I63133 I63139 I6319 I6320 I63211 I63212 I63213 I63219 I6322 I63231 I63232 I63233 I63239 I6329 I6330 I63311 I63312 I63313 I63319 I63321 I63322 I63323 I63329 I63331 I63332 I63333 I63339 I63341 I63342 I63343 I63349 I6339 I6340 I63411 I63412 I63413 I63419 I63421 I63422 I63423 I63429 I63431 I63432 I63433 I63439 I63441 I63442 I63443 I63449 I6349 I6350 I63511 I63512 I63513 I63519 I63521 I63522 I63523 I63529 I63531 I63532 I63533 I63539 I63541 I63542 I63543 I63549 I6359 I636 I638 I6381 I6000 I6001 I6002 I6010 I6011 I6012 I602 I6020 I6021 I6022 I6030 I6031 I6032 I604 I6050 I6051 I6052 I606 I607 I608 I609 I610 I611 I612 I613 I614 I615 I616 I618 I619 |
| Acute Bleed | D62 T82837A T82838A |

**Table S2: Comparison of crude estimates of primary and secondary outcomes with propensity-matched estimates.**

| **Outcomes** | **Crude Estimates** | | | **Propensity Matched Estimates** | | |
| --- | --- | --- | --- | --- | --- | --- |
|  | **TAVR + AFib** | **TAVR Only** | **p** | **TAVR + AFib** | **TAVR Only** | **p** |
| N | 112,945 (28.7%) | 280,250 (71.3%) |  | 21,890 (50.0%) | 21,890 (50.0%) |  |
| In-Hospital Mortality | 1,570 (1.4%) | 3,475 (1.2%) | 0.114 | 306 (1.4%) | 327 (1.5%) | 0.400 |
| STEMI | 2,195 (1.9%) | 4,960 (1.8%) | 0.141 | 422 (1.9%) | 429 (2.0%) | 0.809 |
| Acute Heart Failure | 17,645 (15.6%) | 28,970 (10.3%) | **<0.001** | 3,418 (15.6%) | 2,963 (13.5%) | **<0.001** |
| Ventricular Arrhythmia | 5,315 (4.7%) | 8,970 (3.2%) | **<0.001** | 1,036 (4.7%) | 833 (3.8%) | **<0.001** |
| Cardiogenic Shock | 2,830 (2.5%) | 4,850 (1.7%) | **<0.001** | 550 (2.5%) | 371 (1.7%) | **<0.001** |
| MCS | 1,170 (1.0%) | 2,535 (0.9%) | 0.110 | 225 (1.0%) | 262 (1.2%) | 0.092 |
| MACE | 5,660 (5.0%) | 11,485 (4.1%) | **<0.001** | 1,099 (5.0%) | 981 (4.5%) | **0.008** |
| AKI | 14,330 (12.7%) | 23,495 (8.4%) | **<0.001** | 2,769 (12.7%) | 2,275 (10.4%) | **<0.001** |
| Acute Stroke | 1,565 (1.4%) | 3,580 (1.3%) | 0.267 | 300 (1.4%) | 243 (1.1%) | **0.014** |
| Acute Bleed | 14,430 (12.8%) | 29,400 (10.5%) | **<0.001** | 2,801 (12.8%) | 2,756 (12.6%) | 0.518 |

*Abbreviations: TAVR, transcatheter aortic valve replacement, Afib, atrial fibrillation, STEMI, ST elevated myocardial infarction, MCS, mechanical circulatory support, MACE, major adverse cardiac events, AKI, acute kidney injury*

**Table S3: Multivariate analysis of primary and secondary outcomes assessing the effect of Afib in TAVR patients compared to those TAVR patients without Afib**

| **Outcome** | **Adjusted OR** | **95% CI** | **p** |
| --- | --- | --- | --- |
| In-Hospital Mortality | 0.99 | 0.85-1.15 | 0.907 |
| STEMI | 1.02 | 0.89-1.17 | 0.777 |
| Acute Heart Failure | 1.27 | 1.20-1.34 | **<0.001** |
| Ventricular Arrhythmia | 1.33 | 1.22-1.45 | **<0.001** |
| Cardiogenic Shock | 1.38 | 1.23-1.56 | **<0.001** |
| MCS | 1.07 | 0.89-1.29 | 0.456 |
| MACE | 1.14 | 1.05-1.24 | **0.002** |
| AKI | 1.27 | 1.20-1.35 | **<0.001** |
| Acute Stroke | 1.07 | 0.92-1.25 | 0.371 |
| Acute Bleed | 1.20 | 1.13-1.27 | **<0.001** |

*Abbreviations: TAVR, transcatheter aortic valve replacement, Afib, atrial fibrillation, aOR, adjusted odds ratio, CI, confidence interval, STEMI, ST elevated myocardial infarction, MCS, mechanical circulatory support, MACE, major adverse cardiac events, AKI, acute kidney injury*

**Table S4: Variables adjusted for in the logistic regression model and propensity-score matching.**

| Age |
| --- |
| Sex |
| Race |
| Hyperlipidemia |
| Hypertension |
| Chronic Heart Failure |
| Prior MI |
| Prior PCI |
| Prior CABG |
| Prior Pacemaker |
| Obesity |
| Smoker/Nicotine user |
| Chronic Obstructive Pulmonary Disease |
| OSA |
| Pulmonary Hypertension |
| Prior Stroke |
| Liver Disease |
| CKD/ESRD |
| Diabetes Mellitus |
| Hypothyroidism |
| Nutritional Anemia |
